# Who is seeking help for psychological distress associated with the COVID-19 pandemic? Characterization of risk factors in 1269 participants accessing low-threshold psychological help

**DOI:** 10.1371/journal.pone.0271468

**Published:** 2022-07-18

**Authors:** Kevin Hilbert, Ole Boeken, Julia Asbrand, Sophia Seemann, Till Langhammer, Berit Praxl, Leonore Horváth, Andrea Ertle, Ulrike Lueken

**Affiliations:** Department of Psychology, Humboldt-Universität zu Berlin, Berlin, Germany; Public Library of Science, UNITED STATES

## Abstract

**Background:**

The COVID-19 pandemic and accompanying restrictions are associated with substantial psychological distress. However, it is unclear how this increased strain translates into help-seeking behavior. Here, we aim to characterize those individuals who seek help for COVID-19 related psychological distress, and examine which factors are associated with their levels of distress in order to better characterize vulnerable groups.

**Methods:**

We report data from 1269 help-seeking participants subscribing to a stepped-care program targeted at mental health problems due to the COVID-19 pandemic. Sample characteristics were compared to population data, and linear regression analyses were used to examine which risk factors and stressors were associated with current symptom levels.

**Results:**

Seeking for help for COVID-19 related psychological distress was characterized by female gender, younger age, and better education compared to the general population. The majority reported mental health problems already before the pandemic. 74.5% of this help-seeking sample also exceeded clinical thresholds for depression, anxiety, or somatization. Higher individual symptom levels were associated with higher overall levels of pandemic stress, younger age, and pre-existing mental health problems, but were buffered by functional emotion regulation strategies.

**Conclusions:**

Results suggest a considerable increase in demand for mental-healthcare in the pandemic aftermath. Comparisons with the general population indicate diverging patterns in help-seeking behavior: while some individuals seek help themselves, others should be addressed directly. Individuals that are young, have pre-existing mental health problems and experience a high level of pandemic stress are particularly at-risk for considerable symptom load. Mental-healthcare providers should use these results to prepare for the significant increase in demand during the broader aftermath of the COVID-19 pandemic as well as allocate limited resources more effectively.

## Introduction

Soon after the emergence of COVID-19 and its pandemic spreading, first reports indicated that this disease would not only cause a medical but a mental health crisis as well [1, 2]. A subsequent, considerable corpus of work demonstrated an increase in general psychological distress and a wide range of symptoms including anxiety and depression due to the pandemic’s burden (as reviewed in [3]). Overall, a recent meta-regression estimated a global increase of 27.6% and 25.6% for major depressive disorder and anxiety disorders, respectively [4].

It is crucial to point out, that many mental health problems will occur with considerable delay and a substantial share of affected individuals may experience a relatively chronic course [5–7]. This indicates an increase in the need for mental healthcare in the near future and indeed, first reports seem to confirm this notion: in Germany, a survey conducted by a professional body of psychotherapists reported an increase in demand for an initial psychotherapy session of 40.8% in January 2021 compared to January 2020 [8].

Following diathesis-stress models, individual burden and symptoms emerges by the interplay between situational stressors and pre-existing vulnerability which can be modified by the ability to successfully regulate emotions [9]. For the COVID-19 pandemic, first results suggest that duration of quarantine and–more broadly–social isolation, financial difficulties as well as uncertainty may be central stressors [10, 11]. Further, female sex, younger age, low education level and unemployment, and pre-existing mental disorders may be personal dispositions signaling increased risk [11–15]. However, it remains unclear who will seek help for mental problems associated with the pandemic. Therefore, reports on generally increased distress and mental health problems due to the COVID-19 pandemic need to be complemented by data describing which individuals actually seek help. A characterization of such help-seeking samples, including a comparison to representative samples, might shed light on specifics of help-seeking individuals. Furthermore, an examination of which factors help-seeking individuals associate with their levels of distress may be informative on further characterizing vulnerability factors.

Such information will help to allocate limited resources in mental healthcare efficiently and to plan for upcoming treatment-demand. Accordingly, we used baseline data from an help-seeking sample accessing a stepped-care program designed to alleviate psychological distress due to the COVID-19 pandemic. We examined sociodemographic characteristics, the presence of a range of risk-factors as well as stressors. In addition, we compared core characteristics with population scores from government data and representative studies in Germany in order to characterize this help-seeking sample. We examined which characteristics were associated with symptom severity at the start of this program in order to examine which factors they associate with their levels of distress. We hypothesized that the help-seeking sample will be predominantly female and well-educated. We also expected that their level of distress will be associated with pre-existing psychopathology and that they have had more exposure to adverse experiences in the pandemic. In contrast, high levels of social support and good emotion regulation abilities were expected to be buffering factors.

## Materials and methods

### Trial design

Here, we report cross-sectional data from a sample of help-seeking individuals participating in a stepped-care program against psychological distress due to the COVID-19 pandemic at study-entry (‘baseline’), before any interventions were conducted.

The overall study was planned as a longitudinal observational study following participants over the course of two years [16]. A stepped-care program including a first online intervention and, if necessary, a second face-to-face digital group program was offered. After completing a baseline assessment at study entry, participants were invited to use the online intervention (chatbot program ‘Aury’) which was specifically adapted to the pandemic. Aury provided information and low-threshold interventions for symptoms (e.g. sleeping disorders, depression, anxiety, rumination, interpersonal conflicts) and activated individual resources. After a four week-waiting period, participants with lasting symptoms entered the second step of a 6-week group-based prevention program conducted by cognitive behavioral therapists. Participants were invited for 6 and 12 month follow-ups (still ongoing). All procedures contributing to this work comply with the ethical standards of the relevant national and institutional committees on human experimentation and with the Helsinki Declaration of 1975, as revised in 2013. The study protocol was registered at the German Clinical Trial Register (DRKS00023220; https://www.drks.de) and approved by the ethics committee of the Department of Psychology at Humboldt-Universität zu Berlin (#2020–35).

### Sample

The stepped-care program (“Stressfrei nach Corona: ein psychologisches Hilfsprogramm der Humboldt-Universität zu Berlin”: www.corona-stressfrei.de) was promoted via official press releases and mailing lists of the Humboldt-Universität zu Berlin, social media (Instagram, Facebook). We further dispatched information material to mental health care providers and email contact to Corporate Health Management offices in the Berlin metropolitan area. Additionally, the program was promoted on local and nationwide newspaper, radio and TV interviews.

We examined the baseline data of adults aged 18 or older registering for the stepped-care program from the beginning of the program, September 1, 2020 until the end of the recruitment phase, April 15, 2021. There were n = 1752 registrations of which n = 421 were excluded as incomplete. N = 62 were excluded as double registrations, resulting in a final analysis sample of n = 1269 participants. For those who started or completed the baseline assessment more than once, we selected the earliest complete dataset. We followed the principles of the Declaration of Helsinki, whereby all subjects gave their written informed consent for participation, data collection, analysis and publication.

### Materials and procedures

Participants signed up to the study by providing their email-address to a double-opt-in procedure, and in turn received a link to the baseline assessment. The email-address was used to re-contact participants over the course of the study but was kept separate of any other information. During the baseline assessment, participants generated an individual code for data pseudonymization. They provided sociodemographic information such as age, sex, children and education. Additionally, participants completed the following questionnaires before receiving a link to the first step of the stepped-care program.

#### Patient Health questionnaire (PHQ)

Symptom severity was assessed using three subscales from the PHQ [17]: the PHQ-9 for depressive symptoms, the GAD-7 for anxiety symptoms and the PHQ-15 for somatization. These scales have demonstrated good psychometric properties [17–20], higher scores equal higher symptom load.

#### Short-form-8 (SF-8)

The SF-8 [21] was used to assess health-related quality of life as a secondary outcome. It assesses impairment due to and satisfaction with mental and physical wellbeing with good psychometric properties [21]. Higher scores denote more impairment and lower quality of life.

#### COVID-19 pandemic events questionnaire

A customized collection of items on potential psychological distress associated with the COVID-19 pandemic was developed in-house after the beginning of the pandemic. Items were created based on literature on psychological distress due to natural disasters, previous pandemics, social isolation and quarantine. Items cover distress due to fear and worry about somatic health, due to social isolation and contact restrictions, due to financial or work-related burdens, due to increased child-care or homeschooling, and due to previous mental health problems. Items focused on the first wave of the COVID-19 pandemic in Germany. Additionally, the presence or absence of a variety of emotional states was checked for the time of the first wave of the COVID-19 pandemic in Germany and for the time of the baseline assessment. All items are given in S1 Table.

#### Emotion Regulation Questionnaire (ERQ)

The ERQ [22] was used to assess emotion regulation as a form of coping. It assesses the strategies reappraisal and suppression and showed good psychometric properties.

### Analysis

Data were screened for improbable scores and outliers, both of which were set to missing values, some variables were recoded (details are given in S1 File). We calculated a sum score on the overall level of pandemic stress based on individual items on situation stressors with higher scores indicating more stress (for details see also S1 File). Descriptive statistics were used to examine the sample in detail. We used McNemar’s test to examine whether affective states were currently being reported more frequently than during the first wave of the COVID-19 pandemic. Results were Bonferroni-corrected to account for the high number of affective states.

Comparisons of sample characteristics to population data were done using one-sample t-tests and one-sample chi-square tests as appropriate. Population data was drawn from census data, census extrapolations and representative surveys provided by the German Federal Statistical Office, from statistics by the German Federal Employment Agency and the Robert Koch Institute, which is the German body for monitoring the COVID-19 outbreak (see S3 Table for a detailed description of this data and all data sources).

Separate multiple stepwise linear regression analyses were used in order to examine which risk factors and stressors were associated with mental health problems as indicated by the PHQ-9, GAD-7 and PHQ-15 scales. We used forward selection and decided on a threshold of p < 0.01 for inclusion given the high number of potentially associated variables. All analyses were conducted in SPSS 26 (IBM, Armonk, NY, USA), with the level of significance set at p < 0.05.

## Results

### Overall description of help-seeking sample

S2 Table provides a detailed overview on the raw data for all sociodemographic characteristics and responses to all potential stressors. The help-seeking sample of participants was mainly female (79.4%), well educated (87.1% with highest secondary school or better) and only rarely unemployed (6.6%). Slightly more than half of the participants were in a relationship and slightly less than half of the participants had a child or children. When examining responses to the potential COVID-19 related stressors, marked differences between mean ratings appeared: while participants were less concerned about their individual COVID-19 related risk, they worried substantially about potential threat to friends and relatives. Contact restrictions were perceived as the strongest stressor, while experiencing a burden at work because of the pandemic was common as well. On the contrary, financial worries were rather uncommon in the sample.

**Fig 1:**
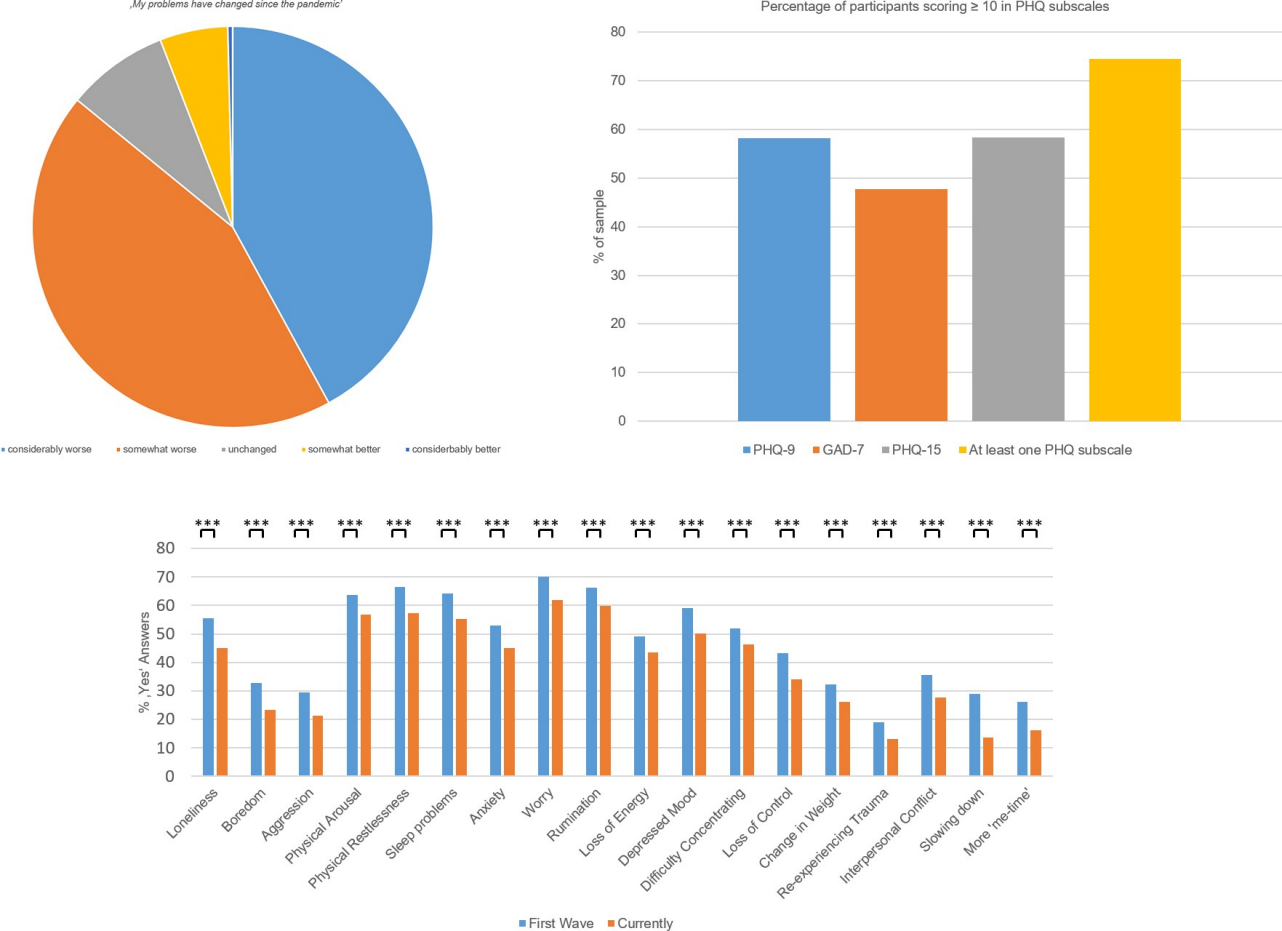
Ratings of psychological distress and clinical scores. Change in mental health problems due to the pandemic in the sample (valid answers only, without N/As; upper). Percentage of participants scoring ten or above across the primary outcomes (middle). Percentage of affective states reported as occurring during the first wave of the COVID-19 pandemic and currently (all categories negative except for the right outer two, lower). Here, all comparisons between both time-points were highly significant (*** p<0.001, all Bonferroni-corrected).

Regarding their mental health, the majority of participants (54.5%) reported having mental health problems before the pandemic already. S1 Fig shows the results from coding the previous mental health problems participants reported in their free responses, indicating that subclinical and clinical anxiety and depression were by far the most common. Crucially, the vast majority of participants with previous mental health problems reported a further deterioration of their mental health due to the pandemic, only few participants reported benefits or no changes (Fig 1). Moreover, a majority of 59.3% of participants reported new mental health problems caused by stress due to the pandemic. For both old and new mental health problems, the lack of support was more common than the availability of support.

The severity of depression and somatization was rated higher than the severity of worry symptoms (means and standard deviations: PHQ-9: 11.3 (5.7), GAD-7: 9.7 (5.0), PHQ-15: 11.3 (5.4)). On all three scales, substantial proportions of participants scored ten or above, which is commonly used as cut off [17]: PHQ-9: 58.2%, GAD-7: 47.7%, PHQ-15: 58.3% (Fig 1). 74.5% scored ten or above in at least one of these scales. Impaired quality of life as measured by the SF-8 was substantial (mean and standard deviation: 23.7 (5.9)). A variety of additional symptoms were reported, with worry, rumination, physical arousal, restlessness and sleep problems being the most frequent ones (Fig 1). Positive experiences such as life slowing down were rare in the sample. All symptoms and experiences were more frequent during the first wave of the pandemic compared to the current assessment which took place during the second to third wave.

### Comparison of help-seeking sample with population data

The participating sample differed in many characteristics from population averages. Participants were considerably more female, younger, and better educated than the general population (see Table 1). No differences to the population emerged when examining the average size of the flat or house. Being unmarried and separated was more frequent, being married, divorced or widowed less frequent in the participating sample compared to the population. More participants reported on having two children or children in general at home and fewer reported on having no children at home, compared to the general population. Substantially more participants worked in the health sector than common for the population, while fewer participants reported a verified COVID-19 infection.

**Table 1:** Comparison of sample characteristics to population values, numbers per category except where noted.

		Sample		Population		Comparison	
		*mean / N*	*sd / %*	*mean / N*	*sd / %*	Chi-square / t	p
*sex*						456.348	<0.001
	female	1008	(79.4)	42,052,500	(50.7)		
	male	238	(18.8)	40,966,700	(49.3)		
*age*		42.1	(13.3)	44.4	N/A	-6.099	<0.001
*education status*						1631.960	<0.001
	lowest secondary school	19	(1.5)	N/A^1^	(29.6)		
	inter-mediate secondary school	144	(11.3)	N/A^1^	(29.9)		
	highest secondary school (including university degree and PhD)	1083	(85.3)	N/A^1^	(32.5)		
	other	23	(1.8)	N/A^1^	(7.8)		
*relationship status*						141.151	<0.001
	unmarried	692	(55.4)	34,661,000	(41.9)		
	married	433	(34.1)	35,351,000	(42.7)		
	separated	44	(3.5)	1,725,000	(2.1)		
	divorced	61	(4.8)	5,727,000	(6.9)		
	widowed	18	(1.4)	5,321,000	(6.4)		
*working in the health sector*						35.598	<0.001
	yes	193	(16.2)	3,657,135	(10.8)		
	no	1001	(83.8)	30,134,730	(89.2)		
*number of children at home*						35.224	<0.001
	none	853	(67.3)	N/A^1^	(72.1)		
	one	182	(14.4)	N/A^1^	(14.2)		
	two	192	(15.2)	N/A^1^	(10.2)		
	three or more	40	(3.2)	N/A^1^	(3.5)		
*housing space (in m^2^)*		93.4	(48.8)	94.1	N/A	-0.547	0.585
*COVID-19 status*						5.078	0.024
	without COVID-19 diagnosis	1217	(97.5)	80,081,589	(96.3)		
	with COVID-19 diagnosis	31	(2.5)	3,073,442	(3.7)		

^1^ Absolute numbers for the population were not available, but percentage data was. Chi-square tests were done by comparing the actual number of participants per category in the sample with the expected number of participants given the population percentages. Categories are not raw data, but have been modified in order to match the categories for which population data was available. This means that some categories were dropped for this comparison (such as the *diverse* category for *gender*) while others have been recoded (such as the *highest secondary school category*, which here includes also the *university degree* and *PhD* entries that had their own categories in the raw data). More detail is available in supplement D.

### Contribution of vulnerability factors and pandemic-related stressors on symptom severity

Multiple stepwise linear regression analyses on each PHQ subscale explained substantial proportions of the outcome variance (PHQ-9: R^2^_corr_ = 0.40, GAD-7: R^2^_corr_ = 0.34, PHQ-15: R^2^_corr_ = 0.30). Table 2 presents the associated variables for each regression with their standardized coefficients. General and specific patterns emerged for the associated variables. The overall level of pandemic stress and pre-existing mental health problems were the most important variables associated with increasing symptom severity for all primary outcomes. Moreover, the development of new mental health problems due to the pandemic was associated with higher symptom severity as well. Pre-existing depressiveness and pre-existing depressive disorders were associated with PHQ-9 severity, while the presence of multiple pre-existing mental health problems and younger age were associated with PHQ-9 severity and GAD-7 severity. Worries about not getting adequate medical care for other conditions were associated with PHQ somatization. Interestingly, higher reappraisal in emotion regulation as assessed by the ERQ was associated with lower PHQ-9 and GAD-7 scores.

**Table 2:** Final stepwise regression models on the PHQ scores.

	Coefficient			Modell	
Variable	Standardized Beta	T	p	R	R^2^_corr_
*PHQ-9 Depression*					
Overall level of pandemic stress	0.243	7.138	<0.001		
Suffering from mental health problems before the pandemic	0.169	5.693	<0.001		
ERQ: reappraisal sum score	-0.154	-6.855	<0.001		
Developed mental health problems due to pandemic: yes	0.145	6.181	<0.001		
Mental-health problems before pandemic: Depressive Disorder	0.140	5.306	<0.001		
Found work meaningful or relevant during the pandemic	-0.099	-4.135	<0.001		
ERQ: suppression sum score	0.109	4.884	<0.001		
Age	-0.101	-4.366	<0.001		
Impression that social relationships have suffered long-term damage	0.116	4.365	<0.001		
Relationship status: married	-0.065	-2.818	0.005		
Mental-health problems before pandemic: depressiveness	0.088	3.786	<0.001		
Relationship status: separated	0.069	3.090	0.002		
Mental health problems have changed since the pandemic*	-0.081	-2.834	0.005		
Worried about financial situation	0.132	3.820	<0.001		
Financial losses due to the pandemic	-0.090	-2.798	0.005		
Verified COVID-19 illness: yes	0.058	2.630	0.009		
Mental-health problems before pandemic: multiple problems	0.065	2.591	0.010		
				0.639	0.401
*PHQ GAD-7*					
Overall level of pandemic stress	0.356	11.515	<0.001		
Suffering from mental health problems before the pandemic	0.197	8.245	<0.001		
Developed mental health problems due to pandemic: no	-0.113	-3.265	0.001		
ERQ: reappraisal sum score	-0.176	-7.536	<0.001		
Age	-0.117	-5.053	<0.001		
Mental-health problems before pandemic: multiple problems	0.096	4.078	<0.001		
Developed mental health problems due to pandemic: yes	0.126	3.655	<0.001		
Burden felt at work because of the pandemic	-0.091	-3.050	0.002		
				0.586	0.339
*PHQ-15 Somatoform*					
Overall level of pandemic stress	0.341	11.717	<0.001		
Suffering from mental health problems before the pandemic	0.140	5.530	<0.001		
Had to work from home	-0.081	-3.188	0.001		
Developed mental health problems due to pandemic: yes	0.112	4.514	<0.001		
Worried about not getting adequate medical care for other conditions	0.108	3.987	<0.001		
Occupational status: self-employed	-0.080	-3.332	<0.001		
Sex: male	-0.100	-4.100	<0.001		
Mental-health problems before pandemic: Depressive Disorder	0.084	3.355	<0.001		
Education: PhD	-0.096	-3.905	<0.001		
Worried about having Covid-19, but no test was done or not done in time: no, not worried	-0.071	-2.973	0.003		
Education: university degree	-0.068	-2.691	0.007		
ERQ: suppression sum score	0.064	2.620	0.009		
				0.555	0.308

Total n = 1261.

PHQ-9: Patient Health Questionnaire-9 depression scale; GAD-7: Generalized Anxiety Disorder Scale-7; PHQ-15: Patient Health Questionnaire-15 somatic symptom scale; ERQ: Emotion Regulation Questionnaire.

*Change to the worse coded with lower numbers and change to the better coded with higher numbers, i.e. subjective change to the worse predicted elevated PHQ-9 scores.

## Discussion

Plenty of studies reported increased psychological distress and mental health problems associated with the COVID-19 pandemic for considerable proportions of the population. Yet, it is unclear which individuals will subsequently seek help for these problems and which factors are central drivers of distress in such individuals. Examining a help-seeking sample of 1269 participants from an easily-accessible stepped-care program against COVID-19-induced psychological distress, the extent of depression and anxiety symptoms in our help-seeking sample was substantial with 74.5% of participants scoring above at least one of the cutoff scores, thus indicating a clinically relevant symptom load. We found support for our hypothesis that particularly well-educated women seek psychological support, however, the help-seeking sample also differed from the general population on additional dimensions such as being younger, less frequently married and more having children at home. Partly confirming our second hypotheses, individual psychological distress in this help-seeking sample was associated with pre-existing psychopathology and more exposure to adverse experiences in the pandemic, while emotion regulation (reappraisal) showed a buffering effect.

In line with our expectations, help-seeking individuals as identified by participating in our program were considerably more often female and well-educated. Beyond our hypotheses, we also found the study participants to be younger, more often unmarried and with children at home, more often working in healthcare and less often personally affected by a verified COVID-19 infection than in the general population. Results on sex and age match previous reports on groups who are at increased risk for psychological distress due to the pandemic [11–13]. However, other results stand in contrast to these reports: high education status, for instance, has rather been reported as a protective factor. Note that these results are likely also influenced by diverging patterns in help-seeking behaviors. For instance, younger, female and better educated individuals are also overrepresented in routine psychotherapy compared to their proportion in the general population of Germany An important implication of this finding is that those individuals who seek help for their COVID-19 related psychological distress by themselves are most likely only a portion of all affected individuals. Individuals from certain groups such as those with low educational status may be particularly hard to reach. As such, they may benefit from a broader public health approach addressing them more directly in order to mitigate the individual burden and societal cost of the pandemic’s effect on mental health. Overall, it is important to note that three quarters of our help-seeking sample showed symptoms of anxiety and depression to clinically relevant degree. This considerably exceeds the percentages above cut-offs in the general population (ca. 6.6% for the PHQ-9, 5.1% for the GAD-7, and 9.3% for the PHQ-15; [23–25]). These figures underscore the unmet need for people suffering from mental health problems in the aftermath of the pandemic. Expecting a certain amount of these persons to request professional help is in line with reports from German professional organizations of a more than 40% increase in demand for regular psychotherapy [8]. In summary, this data suggests considerable unmet needs in mental-healthcare during the aftermath of the COVID-19 pandemic. Therefore, a call for low-threshold and scalable psychological interventions for indicated prevention or early intervention in this target group seems warranted. A number of risk factors and stressors were associated with individual symptom severity within this sample of help-seeking individuals. The results indicate that diathesis-stress models of psychological distress also apply to the unique pandemic situation: symptom levels were strongly influenced by the overall level of pandemic stress and by the occurrence of relevant risk factors. Some findings are particularly notable. In agreement with previous research [14, 15], our data indicates that individuals with pre-existing mental health problems are a major high-risk group for psychological distress due to the pandemic and therefore should be monitored closely if possible. This could occur for instance during general practitioner or psychiatrist visits. Second, reappraisal emotion regulation abilities were found to be important buffers of symptom levels, although this was only true for depression and anxiety but not for somatization. As expected, reappraisal as a functional emotion regulation strategy was associated with lower symptom levels, contrary to suppression. This finding may be exploited in order to mitigate the psychological distress due to this pandemic and future similar events by encouraging the use of low-level (digital) interventions targeting individual emotion regulation abilities, which are becoming more and more available and are tested for efficacy [26]. Third, contrary to our hypotheses, the availability of social support for mental health problems was not associated with symptom levels. However, being married was associated with low symptom levels and being separated was associated with elevated symptom levels for depression. This finding may signify the lack of availability of some sort of support in close relationships. Thus, relationship status may be another sociodemographic factor associated with increased risk for high psychological distress from the disorder. In addition, young age was associated with elevated symptom levels across all PHQ subscales except somatization.

The current investigation complements previous surveys with data from a help-seeking sample of substantial size. The sample is well characterized regarding a variety of sociodemographic characteristics and pandemic-related stressors, enabling both a comparison to representative population data and the explanation of considerable proportions of variance in individual symptoms levels. However, there are also limitations. While the study was advertised on national newspapers and television occasionally, recruitment largely focused on the Berlin metropolitan area. Although we did not record the place of residence for our participants, it seems likely that ours is a relatively urban sample. This may influence some findings such as the low average age of the sample. Additionally, the assessment of pandemic-related stressors focused on the circumstances during the first major lockdown in Germany. It is possible that further important factors affecting individual symptom levels occurred afterwards, or that participants deviated from this focus in their answers. Finally, when comparing the sample to representative population data, both sources used different data collection methods and response categories. For instance, ‘diverse’ was not available as a response category for sex in the population data. As a result, we had to compare data which was not completely similar. However, given that significant differences to the population data were very pronounced, this limitation appears to not had strong consequences for the interpretation of results.

In conclusion, we here report the characteristics from a help-seeking sample including more than one-thousand participants applying for of a stepped-care program targeting psychological distress due to the COVID-19 pandemic. Supplementing previous research, we found that help-seeking behavior was associated with both risk-factors for pandemic-related psychological distress and established patterns in help-seeking behaviors. This indicates that only a subset of individuals with psychological distress will proactively seek help by themselves, while other vulnerable groups (e.g. low socio-economic status, low education background) may need to be addressed more directly. Individual symptom levels were significantly higher among this sample compared to the general population, suggesting an emerging and possibly increasing gap between supply and demand for mental-healthcare in the pandemic aftermath. Moreover, individual symptom levels clearly follow the diathesis-stress approach: individuals of younger age, who have pre-existing mental health problems and experience a high level of pandemic stress are particularly at-risk for a considerable symptom load. This may in turn manifest in either relapse or new incidence of mental disorders if untreated. In summary, these data suggest considerable unmet needs in mental-healthcare in the aftermath of the COVID-19 pandemic and call for low-threshold and scalable psychological interventions for indicated prevention or early intervention in this target group. Providers of mental-healthcare should use these results to prepare for a significant increase in demand of psychological help in the broader aftermath of the COVID-19 pandemic and to allocate limited resources most effectively.

## Supporting information

S1 Table Items and response options for the in-house developed ‘stressors for mental health associated with the COVID-19 pandemic’ questionnaire. (DOCX)

S2 Table Raw response data for the in-house developed ‘stressors for mental health associated with the COVID-19 pandemic’ questionnaire (except free texts). (DOCX)

S3 Table Data sources and analytic approach for comparison with population data. (DOCX)

S1 Fig Previous mental health problems in the sample (valid answers only, without N/As). (DOCX)

S1 File Outlier screening and recoding. (DOCX)
