# Supplementary material for: Who is seeking help for psychological distress associated with the COVID-19 pandemic? Characterization of risk factors in 1269 participants accessing low-threshold psychological help
Source: PLoS One. 2022 Jul 18;17(7):e0271468. doi: 10.1371/journal.pone.0271468 (PMC9292095; doi:10.1371/journal.pone.0271468)
Supplement: S2 Table — (DOCX) [file pone.0271468.s002.docx]

SUPPLEMENT TO:

**Who seeks help for psychological distress associated with the COVID-19 pandemic? Characterization of risk factors in 1269 participants accessing low-threshold psychological help**

Kevin Hilbert, PhD, Ole Boeken, Julia Asbrand, PhD, Sophia Seemann, Till Langhammer, Berit Praxl, Leonore Horváth, Andrea Ertle, PhD, Ulrike Lueken, PhD

| **S2 Table. Raw response data for the in-house developed ‘stressors for mental health associated with the COVID-19 pandemic’ questionnaire (except free texts).** | | | | |
| --- | --- | --- | --- | --- |
| **Item** | **n** | **(%)** | **m** | **(sd)** |
| *Demographic information* |  |  |  |  |
| How old are you? |  |  | 42.12 | (13.29) |
| What is your gender? |  |  |  |  |
| - Female | 1008 | (79.4) |  |  |
| - Male | 238 | (18.8) |  |  |
| - Diverse | 10 | (0.8) |  |  |
| - Don't know | 3 | (0.2) |  |  |
| - N/A | 10 | (0.8) |  |  |
| What is your marital status? |  |  |  |  |
| - Single | 318 | (25.1) |  |  |
| - in partnership | 374 | (29.5) |  |  |
| - married | 433 | (34.1) |  |  |
| - separated | 44 | (3.5) |  |  |
| - divorced | 61 | (4.8) |  |  |
| - widowed | 18 | (1.4) |  |  |
| - other | 13 | (1.0) |  |  |
| - N/A | 8 | (0.6) |  |  |
| How many children do you have? |  |  | 0.82 | (1.02) |
| How many children live in your household? |  |  | 0.56 | (1.98) |
| How old is your youngest child? |  |  | 13.96 | (11.97) |
| How old is your oldest child? |  |  | 17.34 | (12.93) |
| What are your housing conditions? |  |  |  |  |
| - in flat | 835 | (65.8) |  |  |
| - in house | 366 | (28.8) |  |  |
| - alone | 246 | (19.4) |  |  |
| - with partner | 564 | (44.4) |  |  |
| - with children | 335 | (26.4) |  |  |
| - with parents | 78 | (6.1) |  |  |
| - with flatmates | 104 | (8.2) |  |  |
| - with pet | 262 | (20.6) |  |  |
| - with garden | 403 | (31.8) |  |  |
| - with opportunity to reach  nature | 529 | (41.7) |  |  |
| - N/A | 4 | (0.3) |  |  |
| What size is your home (in square meters)? |  |  | 93.35 | (48.75) |
| What is your highest education degree? |  |  |  |  |
| - None | 1 | (0.1) |  |  |
| - lowest secondary school | 19 | (1.5) |  |  |
| - intermediate secondary school | 143 | (11.3) |  |  |
| - highest secondary school | 329 | (25.9) |  |  |
| - university degree | 685 | (54.0) |  |  |
| - PhD | 58 | (4.6) |  |  |
| - other | 29 | (2.3) |  |  |
| - N/A | 5 | (0.4) |  |  |
| What is your present occupational status? |  |  |  |  |
| - unemployed | 84 | (6.6) |  |  |
| - in training / studying | 194 | (15.3) |  |  |
| - employed | 685 | (54.0) |  |  |
| - self-employed | 148 | (11.7) |  |  |
| - civil servant | 84 | (6.6) |  |  |
| - retired | 102 | (8.0) |  |  |
| - other | 64 | (5.0) |  |  |
| - N/A | 9 | (0.7) |  |  |
|  |  |  |  |  |
| *Physical health*  *What problematic situations have you experienced yourself during the COVID-19 pandemic?*  *Please refer to the period from the beginning of the contact restrictions (approx. Chancellor Angela Merkel's speech on March 18, 2020) to the first major relaxations (approx. Whitsun 2020).* | | | | |
| I was worried about having Covid-19, but no test was done or not done in time. |  |  |  |  |
| - yes | 226 | (17.8) |  |  |
| - worried, but a test was done in time | 218 | (17.2) |  |  |
| - no, not worried | 770 | (60.7) |  |  |
| - N/A | 55 | (4.3) |  |  |
| I was verifiable contracted with Covid-19 myself. |  |  |  |  |
| - yes | 31 | (2.4) |  |  |
| - no | 1217 | (95.9) |  |  |
| - N/A | 21 | (1.7) |  |  |
| If yes: the course of my illness was... |  |  |  |  |
| - mild | 29 | (2.3) |  |  |
| - moderate | 20 | (1.6) |  |  |
| - severe | 4 | (0.3) |  |  |
| - N/A | 1216 | (95.8) |  |  |
| I was worried about not getting adequate medical care for other conditions (e.g. postponing treatment, not getting appointments with my primary care physician...). |  |  |  |  |
| - not at all | 405 | (31.9) |  |  |
| - a little bit | 342 | (27.0) |  |  |
| - somewhat | 287 | (22.6) |  |  |
| - strongly | 125 | (9.9) |  |  |
| - very strongly | 59 | (4.6) |  |  |
| - N/A | 51 | (4.0) |  |  |
| I was worried that relatives or acquaintances are threatened by Covid-19. |  |  |  |  |
| - not at all | 62 | (4.9) |  |  |
| - a little bit | 208 | (16.4) |  |  |
| - somewhat | 332 | (26.2) |  |  |
| - strongly | 356 | (28.1) |  |  |
| - very strongly | 300 | (23.6) |  |  |
| - N/A | 11 | (0.9) |  |  |
| I would assign myself to a risk group. |  |  |  |  |
| - not at all | 568 | (44.8) |  |  |
| - a little bit | 243 | (19.1) |  |  |
| - somewhat | 227 | (17.9) |  |  |
| - strongly | 140 | (11.0) |  |  |
| - very strongly | 80 | (6.3) |  |  |
| - N/A | 11 | (0.9) |  |  |
|  |  |  |  |  |
| *Social isolation and contact restriction*  *What problematic situations have you experienced yourself during the COVID-19 pandemic?*  *Please refer to the period from the beginning of the contact restrictions (approx. Chancellor Angela Merkel's speech on March 18, 2020) to the first major relaxations (approx. Whitsun 2020).* | | | | |
| The contact restrictions have burdened me overall. |  |  |  |  |
| - not at all | 26 | (2.0) |  |  |
| - a little bit | 109 | (8.6) |  |  |
| - somewhat | 308 | (24.3) |  |  |
| - strongly | 464 | (36.6) |  |  |
| - very strongly | 359 | (28.3) |  |  |
| - N/A | 3 | (0.2) |  |  |
| I have the impression that my social relationships, e.g. with friends or family members, have suffered long-term damage. |  |  |  |  |
| - not at all | 235 | (18.5) |  |  |
| - a little bit | 282 | (22.2) |  |  |
| - somewhat | 403 | (31.8) |  |  |
| - strongly | 241 | (19.0) |  |  |
| - very strongly | 99 | (7.8) |  |  |
| - N/A | 9 | (0.7) |  |  |
| I have the impression that I or someone I am closely attached to (partner, children, etc.) have suffered long-term damage due to the contact restrictions. |  |  |  |  |
| - not at all | 189 | (14.9) |  |  |
| - a little bit | 286 | (22.5) |  |  |
| - somewhat | 377 | (29.7) |  |  |
| - strongly | 280 | (22.1) |  |  |
| - very strongly | 126 | (9.9) |  |  |
| - N/A | 11 | (0.9) |  |  |
|  |  |  |  |  |
| *Financial situation*  *What problematic situations have you experienced yourself during the COVID-19 pandemic?*  *Please refer to the period from the beginning of the contact restrictions (approx. Chancellor Angela Merkel's speech on March 18, 2020) to the first major relaxations (approx. Whitsun 2020).* | | | | |
| I suffered financial losses due to the pandemic. |  |  |  |  |
| - not at all | 717 | (56.5) |  |  |
| - a little bit | 171 | (13.5) |  |  |
| - somewhat | 194 | (15.3) |  |  |
| - strongly | 102 | (8.0) |  |  |
| - very strongly | 74 | (5.8) |  |  |
| - N/A | 11 | (0.9) |  |  |
| I was worried about my financial situation. |  |  |  |  |
| - not at all | 544 | (42.9) |  |  |
| - a little bit | 250 | (19.7) |  |  |
| - somewhat | 184 | (14.5) |  |  |
| - strongly | 150 | (11.8) |  |  |
| - very strongly | 133 | (10.5) |  |  |
| - N/A | 8 | (0.6) |  |  |
| I received financial support. |  |  |  |  |
| - not at all | 864 | (68.1) |  |  |
| - a little bit | 117 | (9.2) |  |  |
| - somewhat | 113 | (8.9) |  |  |
| - much | 55 | (4.3) |  |  |
| - very much | 10 | (0.8) |  |  |
| - N/A | 110 | (8.7) |  |  |
|  |  |  |  |  |
| *Family and job*  *What problematic situations have you experienced yourself during the COVID-19 pandemic?*  *Please refer to the period from the beginning of the contact restrictions (approx. Chancellor Angela Merkel's speech on March 18, 2020) to the first major relaxations (approx. Whitsun 2020).* | | | | |
| I had to provide day-care for my child(ren) at home. |  |  |  |  |
| - yes | 331 | (26.1) |  |  |
| - no | 213 | (16.8) |  |  |
| - no children | 642 | (50.6) |  |  |
| - N/A | 83 | (6.5) |  |  |
| I had to educate my child(ren) at home. |  |  |  |  |
| - yes | 216 | (17.0) |  |  |
| - no | 306 | (24.1) |  |  |
| - no children | 654 | (51.5) |  |  |
| - N/A | 93 | (7.3) |  |  |
| If yes:  I rate the support provided by the teaching staff (e.g. regular contact, provision of materials, online courses) with the school grade ... :* |  |  | 3.46 | 1.26 |
| - 1 | 11 | (0.9) |  |  |
| - 2 | 54 | (4.3) |  |  |
| - 3 | 65 | (5.1) |  |  |
| - 4 | 62 | (4.9) |  |  |
| - 5 | 46 | (3.6) |  |  |
| - 6 | 12 | (0.9) |  |  |
| - N/A | 1019 | (80.3) |  |  |
| I have felt overwhelmed with providing day-care/education for my child. |  |  |  |  |
| - not at all | 45 | (3.5) |  |  |
| - a little bit | 69 | (5.4) |  |  |
| - somewhat | 86 | (6.8) |  |  |
| - strongly | 83 | (6.5) |  |  |
| - very strongly | 64 | (5.0) |  |  |
| - no children | 436 | (34.4) |  |  |
| - N/A | 486 | (38.3) |  |  |
| I was worried about my job situation. |  |  |  |  |
| - not at all | 292 | (23.0) |  |  |
| - a little bit | 227 | (17.9) |  |  |
| - somewhat | 248 | (19.5) |  |  |
| - strongly | 218 | (17.2) |  |  |
| - very strongly | 204 | (16.1) |  |  |
| - N/A | 80 | (6.3) |  |  |
| I had to work from home. |  |  |  |  |
| - yes | 490 | (38.6) |  |  |
| - partly | 301 | (23.7) |  |  |
| - no | 347 | (27.3) |  |  |
| - N/A | 131 | (10.3) |  |  |
| I have felt burdened by working from home. |  |  |  |  |
| - not at all | 163 | (12.8) |  |  |
| - a little bit | 139 | (11.0) |  |  |
| - somewhat | 212 | (16.7) |  |  |
| - strongly | 191 | (15.1) |  |  |
| - very strongly | 131 | (10.3) |  |  |
| - N/A | 433 | (34.1) |  |  |
| I work in a healthcare profession. |  |  |  |  |
| - yes | 193 | (15.2) |  |  |
| - no | 1001 | (78.9) |  |  |
| - N/A | 75 | (5.9) |  |  |
| If yes: To which profession do you assign yourself? |  |  |  |  |
| - doctor | 20 | (1.6) |  |  |
| - nursing | 18 | (1.4) |  |  |
| - elderly care | 8 | (0.6) |  |  |
| - therapist | 23 | (1.8) |  |  |
| - psychotherapist | 35 | (2.8) |  |  |
| - other | 246 | (19.4) |  |  |
| - N/A | 919 | (72.4) |  |  |
| If yes: Have you had any direct or indirect interaction with  Covid-19 patients while working? |  |  |  |  |
| - yes | 91 | (7.2) |  |  |
| - no | 326 | (25.7) |  |  |
| - N/A | 852 | (67.1) |  |  |
| Have you experienced any highly stressful situations at work related to the pandemic? |  |  |  |  |
| - not at all | 200 | (15.8) |  |  |
| - a little | 215 | (16.9) |  |  |
| - sometimes | 269 | (21.2) |  |  |
| - often | 176 | (13.9) |  |  |
| - very often | 127 | (10.0) |  |  |
| - N/A | 282 | (22.2) |  |  |
| How much of a burden did you feel in your work because of the pandemic? |  |  |  |  |
| - not at all | 62 | (4.9) |  |  |
| - a little bit | 149 | (11.7) |  |  |
| - somewhat | 280 | (22.1) |  |  |
| - strongly | 358 | (28.2) |  |  |
| - very strongly | 208 | (16.4) |  |  |
| - N/A | 212 | (16.7) |  |  |
| How meaningful or relevant did you find your work during the pandemic? |  |  |  |  |
| - not at all | 227 | (17.9) |  |  |
| - a little bit | 162 | (12.8) |  |  |
| - somewhat | 261 | (20.6) |  |  |
| - strongly | 230 | (18.1) |  |  |
| - very strongly | 141 | (11.1) |  |  |
| - N/A | 248 | (19.5) |  |  |
|  |  |  |  |  |
| *Psychological health*  *What problematic situations have you experienced yourself during the COVID-19 pandemic?*  *Please refer to the period from the beginning of the contact restrictions (approx. Chancellor Angela Merkel's speech on March 18, 2020) to the first major relaxations (approx. Whitsun 2020).* | | | | |
| I was already suffering from mental health problems or a disorder before the pandemic. |  |  |  |  |
| - yes | 691 | (54.5) |  |  |
| - no | 521 | (41.1) |  |  |
| - N/A | 57 | (4.5) |  |  |
| If yes: My problems have changed since the pandemic. |  |  |  |  |
| - very much better | 3 | (0.2) |  |  |
| - slightly better | 42 | (3.3) |  |  |
| - unchanged | 63 | (5.0) |  |  |
| - slightly worse | 336 | (26.5) |  |  |
| - very much worse | 322 | (25.4) |  |  |
| - N/A | 503 | (39.6) |  |  |
| If yes: I have received support for my mental health problems despite the pandemic. |  |  |  |  |
| - yes | 296 | (23.3) |  |  |
| - no | 337 | (26.6) |  |  |
| - N/A | 636 | (50.1) |  |  |
| I developed mental health problems because of stresses caused by the pandemic. |  |  |  |  |
| - yes | 753 | (59.3) |  |  |
| - no | 347 | (27.3) |  |  |
| - N/A | 169 | (13.3) |  |  |
| If yes: Have you found a partner/support for your problems? |  |  |  |  |
| - yes | 359 | (28.3) |  |  |
| - no | 457 | (36.0) |  |  |
| - N/A | 453 | (35.7) |  |  |
| *What symptoms did you generally experience during this period? If none apply, please select N/A. Please refer to the period from the beginning of the contact restrictions (approx. Chancellor Angela Merkel's speech on March 18, 2020) to the first major relaxations (approx. Whitsun 2020). (multiple answers possible)* | | | | |
| - Loneliness | 703 | (55.4) |  |  |
| - Boredom | 418 | (32.9) |  |  |
| - Aggression | 374 | (29.5) |  |  |
| - Physical tension | 807 | (63.6) |  |  |
| - Physical restlessness | 843 | (66.4) |  |  |
| - Sleep problems | 815 | (64.2) |  |  |
| - Anxiety | 673 | (53.0) |  |  |
| - Worry | 888 | (70.0) |  |  |
| - Rumination | 839 | (66.1) |  |  |
| - Loss of energy (e.g. not getting out of bed) | 622 | (49.0) |  |  |
| - Depressiveness | 749 | (59.0) |  |  |
| - Difficulty concentrating | 660 | (52.0) |  |  |
| - Feeling of loss of control | 549 | (43.3) |  |  |
| - Weight change | 410 | (32.3) |  |  |
| - Re-experiencing a trauma situation (e.g., unwanted memories and images, flashbacks, nightmares) | 241 | (19.0) |  |  |
| - Conflicts | 452 | (35.6) |  |  |
| - Slowing down life | 367 | (28.9) |  |  |
| - More time for myself | 333 | (26.2) |  |  |
| - N/A | 13 | (1.0) |  |  |
| *What symptoms are currently still present?* |  |  |  |  |
| - Loneliness | 571 | (45.0) |  |  |
| - Boredom | 296 | (23.3) |  |  |
| - Aggression | 270 | (21.3) |  |  |
| - Physical tension | 720 | (56.7) |  |  |
| - Physical restlessness | 728 | (57.4) |  |  |
| - Sleep problems | 700 | (55.2) |  |  |
| - Anxiety | 571 | (45.0) |  |  |
| - Worry | 787 | (62.0) |  |  |
| - Rumination | 760 | (59.9) |  |  |
| - Loss of energy (e.g. not getting out of bed) | 552 | (43.5) |  |  |
| - Depressiveness | 636 | (50.1) |  |  |
| - Difficulty concentrating | 586 | (46.2) |  |  |
| - Feeling of loss of control | 431 | (34.0) |  |  |
| - Weight change | 332 | (26.2) |  |  |
| - Re-experiencing a trauma situation (e.g., unwanted memories and images, flashbacks, nightmares) | 165 | (13.0) |  |  |
| - Conflicts | 352 | (27.7) |  |  |
| - Slowing down life | 173 | (13.6) |  |  |
| - More time for myself | 207 | (16.3) |  |  |
| - N/A | 28 | (2.2) |  |  |
|  |  |  |  |  |
| *Note:* *german school grade system (1 – best mark, 6 – worst mark) | | | | |
