# Supplementary material for: Who is seeking help for psychological distress associated with the COVID-19 pandemic? Characterization of risk factors in 1269 participants accessing low-threshold psychological help
Source: PLoS One. 2022 Jul 18;17(7):e0271468. doi: 10.1371/journal.pone.0271468 (PMC9292095; doi:10.1371/journal.pone.0271468)
Supplement: S3 Table — (DOCX) [file pone.0271468.s003.docx]

SUPPLEMENT TO:

**Who is seeking help for psychological distress associated with the COVID-19 pandemic? Characterization of risk factors in 1269 participants accessing low-threshold psychological help**

Kevin Hilbert, PhD, Ole Boeken, Julia Asbrand, PhD, Sophia Seemann, Till Langhammer, Berit Praxl, Leonore Horváth, Andrea Ertle, PhD, Ulrike Lueken, PhD

| **S3 table. Data sources and analytic approach for comparison with population data.** | | | | |
| --- | --- | --- | --- | --- |
| Information | Source and year | value | statistical approach | comment |
| sex distribution | 2018 extrapolation of the 2011 census data^1^, table 1.1 | male: 40,966,700  female: 42,052,500  total population: 83,019,200 | one-sample chi-square test | only available for female and male |
| mean age | 2018 extrapolation of the 2011 census data^1^, table 1.5 | mean age: 44.4 | one-sample t-test |  |
| education status | 2018, yearly representative survey of ca. 1% of population [‘micro-census’] and 2011 census data^2^, table 3.1.1 | still visiting school: 3.6%  lowest secondary school: 29.6%  intermediate secondary school: 29.9% (including ‘Abschluss der polytechnischen Oberschule’)  highest secondary school: 32.5%  other: 0.2%  without graduation: 4.0% | one-sample chi-square test | still visiting school + other + without graduation were combined to match our category *other*; our categories *highest secondary school* & *university degree or PhD* were combined to match the category highest secondary school in the population data; population data here only for individuals > 15 years of age |
| relationship status | 2019, yearly representative survey of ca. 1% of population [‘micro-census’] and 2011 census data^3^, table 2.1 | unmarried: 41.9% married: 42.7%  separated: 2.1%  divorced: 6.9% widowed: 6.4% | one-sample chi-square test | our categories *singe* and *in partnership* were combined to match category unmarried in population data; category *other* was dropped |
| working in the health sector | 2020, September count of jobs subject to mandatory security payments^4^, table 1 | medical healthcare jobs and non-medical healthcare-, hygiene- and wellness-jobs: 3,657,135  total jobs: 33,791,865 | one-sample chi-square test |  |
| number of children at home | 2019, yearly representative survey of ca. 1% of population [‘micro-census’]^5^, table 1.1 | none: 29,941 one: 5,898  two: 4,231 three or more: 1,435 (including categories 3 children, 4 children and 5 children and more) | one-sample chi-square test | reported per household |
| housing space (in square meters) | 2018, yearly representative survey of ca. 1% of population [‘micro-census’] and 2011 census data^6^, table 1 | Mean housing space (in square meters): 94.1m² | one-sample t-test |  |
| COVID-19 status | for number of COVID-19 cases: 15.04.2021, total number of cases reported to the national count^7^, sheet ‘Fälle-Todesfälle-gesamt’; for population count: 2020 extrapolation of the 2011 census data for December 31^st^, 2020^8^ | cases: 3,073,442  total population: 83,155,031 | one-sample chi-square test |  |
| Population data was largely drawn from census data, other government statistics and representative studies in Germany. For some information, no suitable population data was found. It is important to note that available information had to be collected from different years and sources. The population data was then either used to calculate expected values for one-sample chi-square tests or as expected value in a one-sample t-test, as appropriate. Supplemental table 3 provides an overview on which data was used, from what source and year it was collected, and how it was used for statistical comparisons.  1 Statistisches Bundesamt (Destatis). (2020). *Bevölkerung und Erwerbstätigkeit: Bevölkerungsfortschreibung auf Grundlage des Zensus 2011* (Publication No. 20112010130187005). Retrieved from https://www.destatis.de/DE/Themen/Gesellschaft-Umwelt/Bevoelkerung/Bevoelkerungsstand/Publikationen/Downloads-Bevoelkerungsstand/bevoelkerungsfortschreibung-2010130187005.xlsx?__blob=publicationFile  2 Statistisches Bundesamt (Destatis). (2019). *Statistisches Jahrbuch 2019* (Publication No. 1010110-19700-4). Retrieved from https://www.destatis.de/DE/Themen/Querschnitt/Jahrbuch/jb-bildung.pdf?__blob=publicationFile  3 Statistisches Bundesamt (Destatis). (2020). *Bevölkerung und Erwerbstätigkeit:* *Haushalte und Familien - Ergebnisse des Mikrozensus - Fachserie 1 Reihe 3 – 2019.* (Publication No. 2010300197004). Retrieved from https://www.destatis.de/DE/Themen/Gesellschaft-Umwelt/Bevoelkerung/Haushalte-Familien/Publikationen/Downloads-Haushalte/haushalte-familien-2010300197004.pdf?__blob=publicationFile  4 Bundesagentur für Arbeit. (2021). *Beschäftigte nach Berufen (KldB 2010) (Zeitreihe Quartalszahlen)*. Retrieved from https://statistik.arbeitsagentur.de/Statistikdaten/Detail/Aktuell/iiia6/beschaeftigung-sozbe-kldb2010-zeitreihe/kldb2010-zeitreihe-d-0-xlsx.xlsx  5 Statistisches Bundesamt (Destatis). (2020). *Bevölkerung und Erwerbstätigkeit: Haushalte und Familien – Ergebnisse des Mikrozensus* (Publication No. 2010300197004).  Retrieved from https://www.destatis.de/DE/Themen/Gesellschaft-Umwelt/Bevoelkerung/Haushalte-Familien/Publikationen/Downloads-Haushalte/haushalte-familien-2010300197004.pdf?__blob=publicationFile  6 Statistisches Bundesamt (Destatis). (2019). *Wohnen in Deutschland: Zusatzprogramm des Mikrozensus 2018*.  Retrieved from https://www.destatis.de/DE/Themen/Gesellschaft-Umwelt/Wohnen/Publikationen/Downloads-Wohnen/wohnen-in-deutschland-5122125189005.html  7 Robert Koch Institut (RKI). (2021). *Gesamtübersicht der pro Tag ans RKI übermittelten Fälle, Todesfälle und 7-Tage-Inzidenzen nach Bundesland und Landkreis sowie Archiv Fallzahlen und 7-Tage-Inzidenzen nach Landkreis seit 18.11.2020 (22.4.2021)*. Retrieved from https://www.rki.de/DE/Content/InfAZ/N/Neuartiges_Coronavirus/Daten/Fallzahlen_Kum_Tab.xlsx?__blob=publicationFile  8 Statistisches Bundesamt (Destatis). (2021). *Bevölkerung nach Nationalität und Geschlecht*. Retrieved from https://www.destatis.de/DE/Themen/Gesellschaft-Umwelt/Bevoelkerung/Bevoelkerungsstand/Tabellen/zensus-geschlecht-staatsangehoerigkeit-2020.html | | | | |
