# Supplementary material for: Who is seeking help for psychological distress associated with the COVID-19 pandemic? Characterization of risk factors in 1269 participants accessing low-threshold psychological help
Source: PLoS One. 2022 Jul 18;17(7):e0271468. doi: 10.1371/journal.pone.0271468 (PMC9292095; doi:10.1371/journal.pone.0271468)
Supplement: S1 Fig — (DOCX) [file pone.0271468.s004.docx]

SUPPLEMENT TO:

**Who is seeking help for psychological distress associated with the COVID-19 pandemic? Characterization of risk factors in 1269 participants accessing low-threshold psychological help**

Kevin Hilbert, PhD, Ole Boeken, Julia Asbrand, PhD, Sophia Seemann, Till Langhammer, Berit Praxl, Leonore Horváth, Andrea Ertle, PhD, Ulrike Lueken, PhD

| 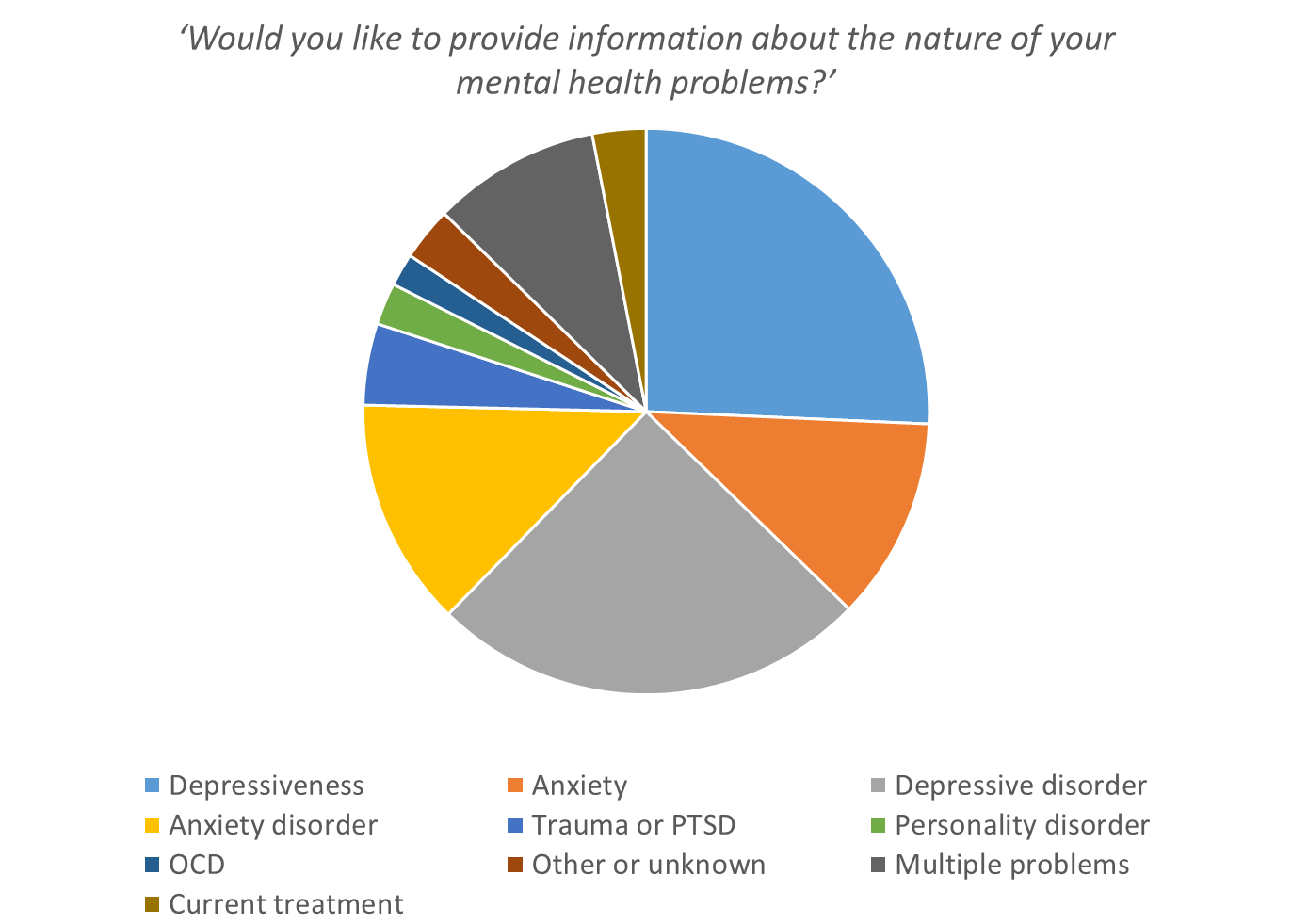 |
| --- |
| **S1 fig.** Previous mental health problems in the sample (valid answers only, without N/As).  This figure shows the results from coding the previous mental health problems participants reported in their free responses (described in S1 File). Depressiveness and Anxiety categories differ from Depressive disorder and Anxiety disorder categories insofar that the latter were assigned if participants indicated a formal diagnosis or treatment. Individual participants were able to report several previous mental health problems. |
