# Supplementary material for: Who is seeking help for psychological distress associated with the COVID-19 pandemic? Characterization of risk factors in 1269 participants accessing low-threshold psychological help
Source: PLoS One. 2022 Jul 18;17(7):e0271468. doi: 10.1371/journal.pone.0271468 (PMC9292095; doi:10.1371/journal.pone.0271468)
Supplement: S1 File — (DOCX) [file pone.0271468.s005.docx]

SUPPLEMENT TO:

**Who is seeking help for psychological distress associated with the COVID-19 pandemic? Characterization of risk factors in 1269 participants accessing low-threshold psychological help**

Kevin Hilbert, PhD, Ole Boeken, Julia Asbrand, PhD, Sophia Seemann, Till Langhammer, Berit Praxl, Leonore Horváth, Andrea Ertle, PhD, Ulrike Lueken, PhD

**S1 File: Outlier screening and recoding**

Frequency data and descriptive statistics were used to screen for improbable and outlier scores. We identified n = 4 participants with an age substantially <18 years (original values: 0, 4, 5 and 5), n = 2 participants with twenty or more children overall / at home (original values: 20, 31, 31) and n = 2 participants with exactly zero square meters of living space. All these values were set to missing.

Variables were also recoded for the regression analyses: N/A and missing values were set to zero value for dimensional variables. Categorial variables were dummy-coded, with one dummy-variable for every response option except N/A, as N/A and missing values were set to zero in all resulting dummy-variables. Some variables were recoded in a specific way for analysis: for *education status*, we checked if text answers for *other* clearly matched available categories, if so, they were re-assigned to these categories. The variable *I have felt overwhelmed with the care/schooling of my child* was split into one dimensional variable with the categories not at all, a little bit, somewhat, much, very much, and one binary dummy variable *no children*.

In order to represent the overall burden of pandemic-related stressors, we additionally calculated a new variable *overall level of pandemic stress*. Here, we summed the values from all items on the dimensional severity of situation stressors. This included 1) *I was worried about not getting adequate medical care for other conditions*, 2) I *was worried that relatives or acquaintances are threatened by Covid-19*, 3) *I would assign myself to a risk group*, 4) *The contact restrictions have burdened me overall*, 5) *I have the impression that my social relationships, e.g. with friends or family members, have suffered long-term damage*, 6) *I have the impression that I or someone I am closely attached to (partner, children, etc) have suffered long-term damage due to the contact restrictions*, 7) *I* *suffered financial losses due to the pandemic, 8) I was worried about my financial situation*, 9) *I have felt overwhelmed with providing day-care/education for my child*, 10) *I was worried about my job situation*, 11) *I have felt burdened by working from home*, 12) *Have you experienced any highly stressful situations at work related to the pandemic*, and 13) *How much of a burden did you feel in your work because of the pandemic*.

The most extensive recoding was done for the variable *Would you like to provide information about the nature of your mental health problems?*. Free text answers were manually decoded into ten subcategories by presence or absence of specific key terms using a binary system (0= no, 1=yes). In a first step, we aimed to categorize subclinical, hence, moderate manifestations on the spectrum of depressiveness and anxiety. The presence of key terms such as “*sleeping problems*”, “*fatigue*”, “*rumination*”, “*dysthymia*”, “*increased* *sadness*”, “*mood swings*”, “*mind wandering*”, as well as increased psychological strain caused by “*social isolation*” and “*increased stress*” levels would lead to the subcategory of **depressiveness**. A crucial prerequisite for this categorization was, however, the absence of key terms such as “*depression*”, “*history of depression*”, and “*depressive disorder*” and/or (if specified) a negative current treatment status. We build the subcategory of **anxiety** in the same way. Again, a negative current treatment status and/or the absence of key terms such as “*anxiety disorder*”, “*generalized anxiety disorder*”, “*specific phobia*” were basic requirements. Key terms were chosen from the anxiety spectrum such as “*panic attacks*”, increased “*anxiety*” levels, constant “*fear of illness*” (e.g., for themselves and/or family members and close relatives), constant “*fear of social relegation*” (e.g., job loss, monetary loss due to the economic uncertainties because of the lockdown) and “*fear of social exclusion*”.

In the next step, we aimed to build subcategories based on participants’ entries displaying more severe or even clinical manifestations of mental health problems. Based on the number of occurrences and the following basic categorization rules, we found patterns in the data leading to the subcategories of the **depressive disorder**, **anxiety disorder**, **trauma & post-traumatic stress disorder**, **personality disorder** and **obsessive-compulsive disorder**. Putative clinical manifestations with only few entries were included in the subcategory **other or unknown** as described below.

Basic rules for the categorization of a putative **depressive disorder** included the usage of relevant key terms such as “*depression*” and “*recurrent depression*” (standalone or among other key terms from the spectrum of anxiety disorders, OCD, personality disorders and PTSD) or (if specified) an affirmative current treatment status due to their depression as well as an entry of an ICD code (ICD-10, F). Accordingly, the same basic rules applied to the categorization for **anxiety disorder**, **trauma and** **PTSD**, **personality disorder** and **OCD**. Key terms for **anxiety disorder** were such as “*generalized anxiety disorder*”, “*specific phobia*” and “*anxiety disorder*”. “*PTSD*”, “*traumatic loss*”, “*traumatic stroke of fate*”, “*history of* *abuse*”, “*traumatic somatic disease* “(e.g., “*cancer*”, “*coronary artery diseases*”, “*stroke*”) would lead to the subcategory of **trauma & PTSD**. The subcategory **personality disorder** included key terms such as “*personality disorder*”, “*borderline disorder*”, “*depended personality disorder*”, “*avoidant personality disorder*”. Key terms such as “*OCD*”, “*obsessive-compulsive disorder*”, “*recurrent compulsions*” were assigned the subcategory of **OCD**.

Entries were coded as **other or unknown** if users stated that they were undergoing psychological or psychiatric treatment, without mentioning the cause. Other mental health problems with only a few occurrences (e.g., “*eating disorder”*, “*psychosomatic disorder*”) were also assigned this subcategory. The subcategory **multiple problems** included multiple participants‘ entries across **depressive disorder**, **anxiety disorder**, **trauma and PTSD**, **personality disorder** and **OCD**.

Finally, **current treatment status** included information about presence/absence of current psychological (e.g., “*psychoanalysis*”, “*behavioral therapy”*) or psychiatric treatment (e.g., “*antidepressant*s”, “*psychotropic medication*”).
